# TNF-α Inhibitors and Other Biologic Agents for the Treatment of Immune Checkpoint Inhibitor-Induced Myocarditis

**DOI:** 10.3389/fimmu.2022.922782

**Published:** 2022-07-01

**Authors:** Xiaohang Liu, Wei Wu, Ligang Fang, Yingxian Liu, Wei Chen

**Affiliations:** Department of Cardiology, Peking Union Medical College Hospital, Chinese Academy of Medical Sciences & Peking Union Medical College, Beijing, China

**Keywords:** cardio-oncology, immune-related adverse events, ICI-induced myocarditis, biologic agents, TNF-alpha inhibitor

## Abstract

With anti-PD-1 antibodies serving as a representative drug, immune checkpoint inhibitors (ICIs) have become the main drugs used to treat many advanced malignant tumors. However, immune-related adverse events (irAEs), which might involve multiple organ disorders, should not be ignored. ICI-induced myocarditis is an uncommon but life-threatening irAE. Glucocorticoids are the first choice of treatment for patients with ICI-induced myocarditis, but high proportions of steroid-refractory and steroid-resistant cases persist. According to present guidelines, tumor necrosis factor alpha (TNF-α) inhibitors are recommended for patients who fail to respond to steroid therapy and suffer from severe cardiac toxicity, although evidence-based studies are lacking. On the other hand, TNF-α inhibitors are contraindicated in patients with moderate-to-severe heart failure. This review summarizes real-world data from TNF-α inhibitors and other biologic agents for ICI-induced myocarditis to provide more evidence of the efficacy and safety of TNF-α inhibitors and other biologic agents.

## 1 Introduction

Immune checkpoint inhibitors (ICIs) restore the immune response of T cells to tumor cells, and immune checkpoint blockade is currently a well-established treatment for a wide range of solid tumors. The approved ICIs on the market include programmed death-1 checkpoint inhibitors (PD-1_i_), PD ligand-1 checkpoint inhibitors (PD-L1_i_), cytotoxic T-lymphocyte-associated protein-4 inhibitors (CTLA-4_i_), and recently approved lymphocyte activation gene-3 inhibitor (LAG-3_i_) (1, 2). Novel immune oncology targets, including T cell immunoglobulin and ITIM domain (TIGIT) (3), T cell immunoglobulin and mucin domain molecule 3 (Tim-3) (4), B and T lymphocyte attenuator (BTLA) (5), CD47 (6), and other molecules, are now under comprehensive and in-depth investigation and are undergoing clinical trials for combination with PD-1/PD-L1 based therapies to overcome the issue of drug resistance and disease progression.

Although these drugs represent a major milestone in the area of cancer treatment, they are associated with a variety of immune-related adverse events (irAEs) that may affect almost all organs, lead to drug discontinuation, and finally mitigate overall therapeutic efficacy (7). Among the affected organs, ICI-induced myocarditis is not the most common type, with an incidence of 0.27% to 1.14% (8, 9). However, ICI-induced myocarditis is one of the most feared irAEs because the mortality rate is approximately 40% (10). According to the recommendations of the American Society of Clinical Oncology (ASCO), European Society for Medical Oncology (ESMO), Society for Immunotherapy of Cancer (SITC), and National Comprehensive Cancer Network (NCCN), steroids are the initial treatment for ICI-induced myocarditis. When patients are refractory or become resistant to steroids, steroid pulse therapy and the addition of immune suppressive agents, including infliximab, mycophenolate, antithymocyte globulin, intravenous immunoglobulin, abatacept or alemtuzumab should be considered. According to the guidelines, infliximab is associated with heart failure and contraindicated in patients with moderate-severe heart failure (11–14).

Biologic agents have been widely used in various autoimmune disorders due to their targeted inhibition of signaling to alleviate the systemic side effects of steroids and improve efficacy. Tumor necrosis factor-α (TNF-α), interleukin-17 (IL-17), interleukin-6 (IL-6), interleukin-4 (IL-4) antibodies and other drugs have been proven useful in treating rheumatoid arthritis (RA), psoriasis, cytokine release syndrome (CRS), atopic dermatitis (AD) and other autoimmune diseases (15–18). ICI-induced myocarditis is also provoked by the excessive activation of the immune system, ASCO, ESMO, SITC and NCCN all have suggested the choice of biological agents. However, the guidelines are based on expert consensus without strong evidence, and as mentioned in the guidelines, these recommendations might be based on the experience of cardiac transplant rejection (11, 12). More importantly, infliximab is contraindicated in patients with moderate-severe heart failure, but is simultaneously recommended in patients with ICI-induced myocarditis, the latter of whom commonly suffer from severe heart failure.

Therefore, we searched for studies focusing on TNF-α antibodies and other biological agents that were prescribed to patients with ICI-induced myocarditis, aiming to provide more clinical information for the selection of biological agents, time of intervention, efficacy and safety.

## 2 TNF-α Antibodies

### 2.1 Cellular Signaling Mediated by TNF-α and Its Receptors

The TNF superfamily consists of 19 ligands and 29 receptors. Among them, TNF-α represents one of the most potent proinflammatory cytokines (19). In humans, TNF-α is present in the membrane form (mTNF-α) or soluble form (sTNF-α). If the extracellular domain of mTNF-α is cleaved by TNF-α cleaving enzyme (TACE; ADAM17), it is released as sTNF-α (20). TNF-α functions by binding to two structurally distinct membrane receptors, TNFR1 or TNFR2. TNFR1, which contains an intracellular death domain (DD), is expressed on almost every cell type. The expression of TNFR2 can be induced and is limited to cells of the immune system, endothelial cells, and nerve cells (21). TNFR1 is mainly activated by sTNF-α, whereas mTNF-α shows greater affinity for TNFR2 (22).

**Figure 1:**
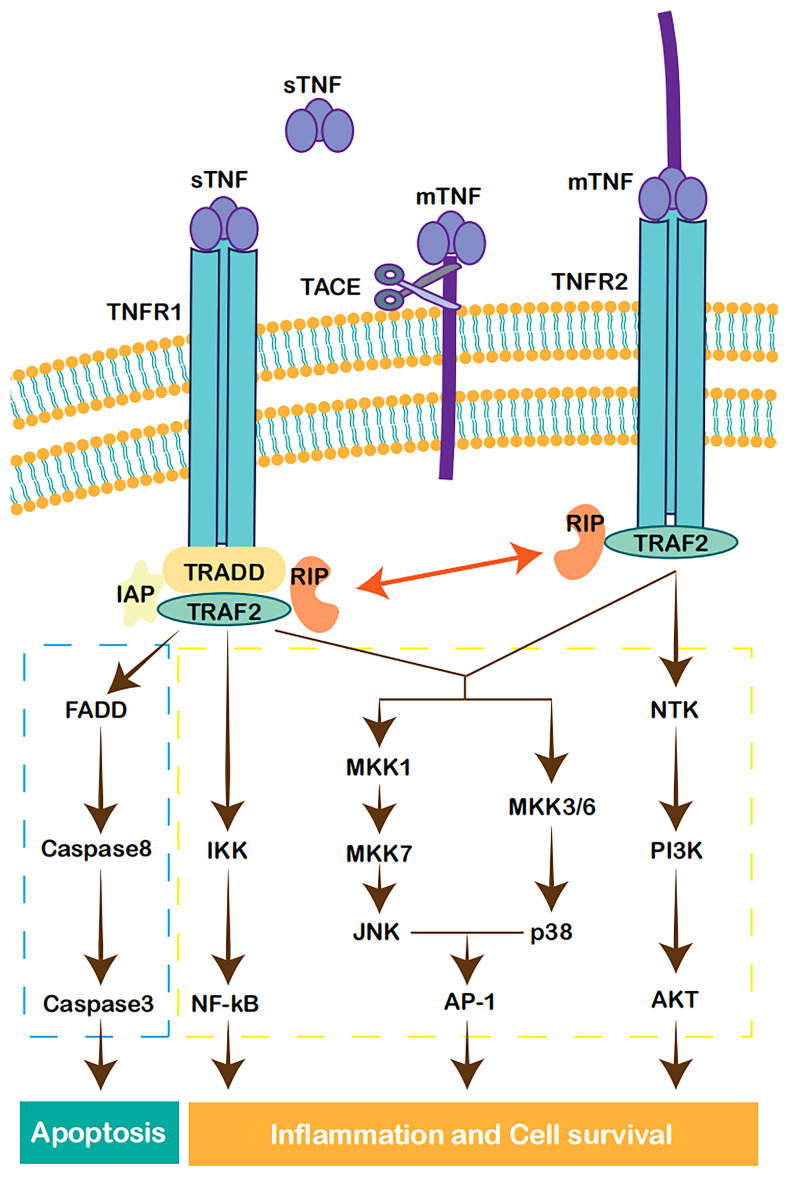
Cell signaling pathways activated by TNF. TNFR1 forms a complex with TRADD, TRAF2, RIP, and IAP, leading to the activation of NF-κB (17). JNK and p38MAPK are also in the downstream of TNFR1 and TRAF2, leading to the activation of AP-1 and other transcription factors (21). The recruitment of TRADD, FADD, and caspase-8, activates caspase-3, which in turn induces apoptosis (22). TNFR2 can also activate MAPK and NF-κB pathways (17, 21). TNFR2 can also realize signal transduction through PI3K/Akt-dependent pathway (23).

The stimulation of TNFR1 or TNFR2 activates distinct cellular signaling responses. After binding to its ligand, TNFR1 recruits TNFR-associated death domain (TRADD), TNF-α receptor associated factor 2 (TRAF2), receptor-interacting protein (RIP) kinase, and inhibitors of apoptosis proteins (IAPs). The TNFR1/TRADD/TRAF2/RIP/IAPs complex activates MAPK- and inhibitor of kappa B kinase (IKK)-dependent pathways. MAPKs, mainly c-Jun N-terminal kinase (JNK) and p38 isoforms, activate activation protein-1 (AP-1) and other transcription factors. IKKs transduce the signal through the activation of NF-κB (19, 23). Alternatively, the sequential recruitment of TRADD, Fas-associated death domain (FADD), and caspase-8 leads to the activation of caspase-3, which ultimately induces apoptosis (24). In comparison, TNFR2 does not contain a DD and is unable to bind TRADD and initiate the subsequent apoptosis pathway. However, without TRADD, TNFR2 also activates the MAPK and NF-κB pathways (19, 23). TNFR2 induces signal transduction through the PI3K/Akt-dependent pathway and modulates downstream effectors (25). The crosstalk between TNFR1 and TNFR2 may be mediated by TRAF2 degradation after prolonged activation, which negatively regulates the pathways of the immune response but enhances TNFR1-induced apoptosis (26, 27). Notably, mTNF-α is capable of reverse signaling mainly *via* TNFR2. Although the mechanism is not well understood, mTNF-α can activate the MAPKs JNK and p38 and downstream transcription factors in the nucleus (28, 29) (**Figure 1**).

### 2.2 Effect of TNF and Its Antagonists on Primary Tumors

TNF was named due to its early promise as a powerful anticancer cytokine, but its role in cancer became quite paradoxical in subsequent research. TNF was first reported to be a factor having the ability to destroy the tumors (30). High doses of TNF caused major destruction of the vascular bed and induced haemorrhagic necrosis in both syngeneic and xenograft tumor models in mice (31, 32). However, systemic TNF injection led to symptoms similar to high doses of endotoxin (32, 33). Therefore, a local approach of isolated limb perfusion (ILP) was created (34). However, TNF does not kill malignant tumor cells directly, and a local TNF injection alone is ineffective (35). TNF must be used in combination with chemotherapy to increase tumor feeding arterial permeability and to increase tissue concentrations of chemotherapy; meanwhile, its metabolic inhibitors inactivate the downstream survival and inflammatory pathways to induce tumor apoptosis (36, 37).

**Figure 2:**
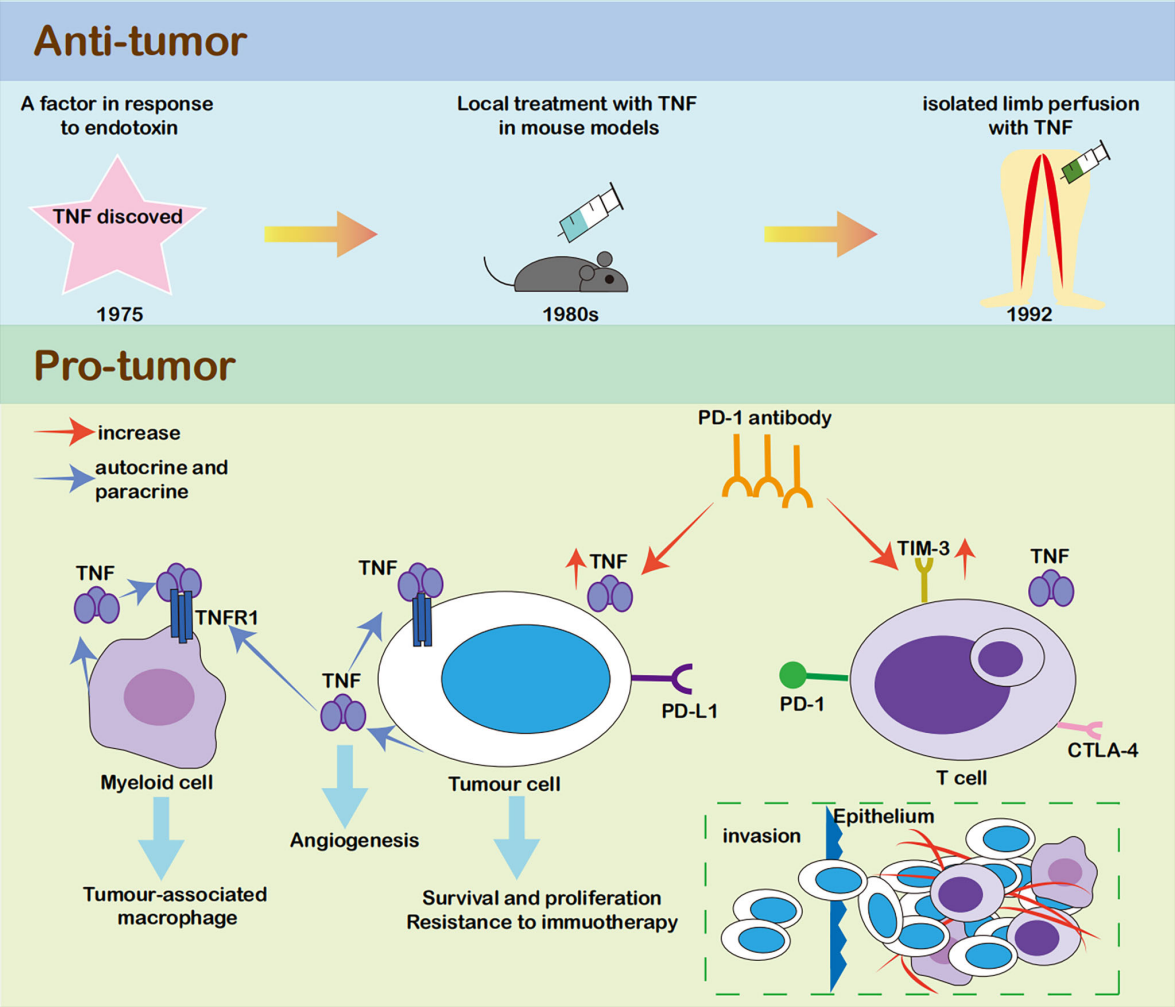
The relationship between TNF and tumour. TNF was first discovered as an anti-tumour factor (28). However, it mainly caused major destruction of the vascular bed rather than killing the malignant cells directly, and its clinical efficacy was limited (29, 32, 33). In recent years, growing evidence showed that both cancer cells and tumour microenvironment produce TNF in an autocrine or paracrine manner. In malignant cells, TNF could cause DNA damage, help survival and proliferation (17, 38). In myeloid cells, TNF led to the generation of tumour-associated macrophage phenotype, which was associated with immune escape and tumour promotion (39, 40). In tumour microenvironment, continuous low concentrations of TNF contributed to angiogenesis, which promoted primary tumor growth and metastases, leukocyte infiltration, and pleural effusion (34, 41). TNF could also induce resistance to chemotherapy (42).

In contrast, accumulating evidence has shown that both cancer cells and the tumor microenvironment produce TNF in an autocrine or paracrine manner. Many cancer cells constitutively secrete picogram quantities of TNF, and host cells, such as myeloid cells, also produce TNF (38, 39). Cancer-related inflammation, of which TNF is a major mediator, promotes tumor development and progression. In malignant cells, TNF causes DNA damage and promotes survival and proliferation (19, 40). In myeloid cells, TNF leads to the generation of a tumor-associated macrophage phenotype, that is associated with immune escape and tumor promotion (41, 42). In the tumor microenvironment, continuous low concentrations of TNF contribute to angiogenesis, which promotes primary tumor growth and metastases, leukocyte infiltration, and pleural effusion (36, 43). TNF also induces resistance to chemotherapy (44) (**Figure 2**). Accordingly, TNF was gradually viewed as a target for cancer treatment. In clinical trials, TNF-α antibodies were not highly effective, while they were safe for use in patients with cancer. Phase I and II clinical trials using the TNF-α antibodies infliximab or etanercept for the treatment of advanced cancer revealed 20% disease stabilization or better response (45–48). Therefore, TNF-α antibodies seem to exert no effect on increasing the risk of tumor growth in patients with cancer.

### 2.3 TNF-α Biologics in the Non-Inflammatory Condition, Ischemic Cardiomyopathy Induced Heart Failure

irAE is a new disease area that has emerged in recent years following the wide use of ICIs (7). Before the appearance of high-grade evidence supported by solid clinical trials, guidelines recommended the use of the TNF-α antibody infliximab to treat ICI-induced myocarditis. However, these guidelines specifically highlight the risk of heart failure caused by TNF-α antibodies (11, 12). This issue is quite interesting and confusing, since patients with ICI-induced myocarditis inevitably have cardiac dysfunction, and moderate-severe heart failure would be common, especially if the patients are at the disease stage when steroids are not sufficient (49). After its first approval by the Food and Drug Administration (FDA) in 1998, clinicians have accumulated a wealth of experience in the use of this TNF-α antibody (50). Therefore, we reviewed the history of TNF-α antibodies in drug development for heart failure and RA as a reference to identify the reason for such a warning in the recommendations and its applicability to ICI-induced myocarditis,.

At the end of the 20^th^ century, the plasma level of TNF-α was found to be elevated in patients with heart failure, and TNF-α contributed to systolic dysfunction, hypertrophy, and myocardial apoptosis in the failing heart (51, 52). Based on these findings, several TNF-α antibodies were initiated in clinical trials as treatments for patients with heart failure. In the Phase I dose escalation trial of etanercept in 18 patients with NYHA class III heart failure, 4 mg/m^2^ and 10 mg/m^2^ etanercept resulted in significant improvements in quality-of-life scores, 6-minute walking distance, and ejection fraction at 14 days after etanercept injection compared to the placebo (53). However, in the Phase III RECOVER and RENAISSANCE trials, etanercept showed no benefit over the placebo in improving the clinical status. The risks of death and chronic heart failure-related hospitalization were even higher. The trials were terminated due to the lack of benefit and potential cardiac safety issues (54). Similarly, in the Phase III ATTACH trial of another TNF-α antibody, infliximab, the percentage of death and heart failure-related hospitalization were not improved, although plasma levels of C-reactive protein (CRP) and IL-6 decreased after each injection. Even in the high dosage group treated with 10 mg/kg infliximab, the risks of death and heart failure-related hospitalization were significantly elevated (55).

Regarding the reason for treatment failure in these trials, the majority of patients with heart failure had ischaemic cardiomyopathy (61%-63% in the RECOVER and RENAISSANCE trials, and 60%-71% in the ATTACH trial) at baseline, and the inflammatory process was likely not the core reason for heart failure in this patient group. Additionally, a physiological dose of TNF-α might exert a protective effect on hypoxic injury in the heart, whereas overinhibition of TNF-α would eliminate this effect (56). Etanercept may prolong the exposure of cardiac tissue to TNF-α by binding to circulating TNF-α and retaining it in the circulation, ultimately leading to cardiac toxicity (57). Similarly, infliximab causes cell lysis in the presence of complement (58). Additionally, a transient but significant increase in TNF-α levels was observed after each infliximab injection, although the authors claimed that the detected TNF-α was not biologically active (55).

### 2.4 TNF-α Biologics in the Inflammatory Condition, RA

In contrast, TNF-α antibodies have been used to treat millions of patients with RA, but their association with cardiovascular events has not been confirmed. Compared to normal subjects, the risk of cardiovascular events is approximately 1.5-2-fold higher in patients with RA (59). According to the US National Data Bank for Rheumatic Diseases, heart failure was significantly less common in anti-TNF-treated patients (2.8%) than in patients treated with disease-modifying antirheumatic drugs (DMARDs) (3.9%) after adjustment for demographic characteristics (60). In a German biologics register, TNF-α inhibitors (etanercept, infliximab, or adalimumab) were shown to be more likely to be beneficial than harmful with regard to the risk of heart failure in patient with RA (61). In another Swedish regional register, the incidence rate of the first cardiovascular disease event was lower in anti-TNF-treated patients (14.0/1000 person-years) than in those who were not treated (35.4/1000 person-years) (62). The results from the British Society for Rheumatology Biologics Register showed a reduction in the incidence of myocardial infarction in patients with RA who responded to anti-TNF-α therapy (3.5/1000 person-years in responders versus 9.4/1000 person-years in nonresponders) (63).

As mentioned above, anti-TNF-α therapy failed in clinical trials and was even associated with a deterioration of heart failure in patients who were mainly diagnosed with ischaemic cardiomyopathy. Therefore, TNF-α antibodies should be administered to patients with ICI-induced myocarditis with caution. However, a similar condition was not observed in patients with RA, in which inflammatory activation might play a more important role in the development of cardiovascular disease, such as ICI-induced myocarditis, and the risk of cardiovascular diseases was not increased after anti-TNF-α therapy (64). We next asked what is the real-world usage of TNF-α antibodies in patients with ICI-induced myocarditis.

### 2.5 TNF-α Antagonists for ICI-Induced Myocarditis

ICI-induced myocarditis is one of the life-threatening irAEs, although it is uncommon. The mortality of this adverse event is approximately 50% (65). ICI-induced myocarditis was reported to occur in 0.06-1.14% of ICI-treated patients. However, subsequent studies suggested that the incidence was likely underestimated because its diagnosis is challenging and data from prospective trials are limited (66). According to previous studies, ICI-induced myocarditis has various manifestations, ranging from an increased serum troponin concentration without obvious symptoms, to chest pain, shortness of breath, arrhythmias, and heart failure (67). The combination of anti-PD-1 and anti-CTLA-4 therapy has been identified as one of risk factors associated with ICI-induced myocarditis (66). Additionally, patients with pre-existing cardiovascular disease tend to suffer more severe ICI-induced cardiovascular toxicity (7). Moreover, patients with ICI-induced myocarditis patients have a higher prevalence of traditional risk factors of cardiovascular disease, such as diabetes and a higher body mass index (9). Genetic background and additional cardiotoxic drugs might also elevate the risk. For instance, germline deletion of Pdcd1 in BALB/c mice results in dilated cardiomyopathy, while C57BL/6 mice with Pdcd1 knockout present no cardiac phenotype (68, 69). The detailed mechanisms of cardiotoxicity caused by ICIs remain to be investigated. The T cell-mediated immune response plays a role in the pathogenesis of ICI-induced myocarditis (70). A histopathological study showed the myocardial infiltration of CD4+ and CD8+ T lymphocytes and macrophages and myocyte death (67). PD-1 and CTLA-4 blockade undoubtedly play important roles in regulating autoimmune responses against myocardium. PD-1^-/-^ mice have diffuse deposition of immunoglobulin G on the surface of cardiomyocytes, causing severe dilated cardiomyopathy and sudden death by congestive heart failure (69). An anti-CTLA-4 antibody also promotes the activation of cardiac-reactive T cells by reducing the number of regulatory T cells that constitutively express CTLA-4 (65). In addition, Johnson and colleagues hypothesized that lymphocytic infiltration of the myocardium might be related to common targeted antigens between cardiac myocytes and tumors. They showed that T cells infiltrating the heart were identical to those in tumors and skeletal muscle using T cell receptor next-generation sequencing (71). To date, management of ICI-induced myocarditis is based on treatment strategies extensively used for other ICI-induced adverse events, including cessation of ICIs, supportive management and glucocorticoids (70). However, steroids alone may not be sufficient for ICI-induced myocarditis. Steroid failure has been classified into steroid-refractory and steroid-resistant. Steroid-refractory was defined as no improvement or aggravation of related symptoms after the initial use of steroids, and steroid-resistant refers to the recurrence of symptoms during steroid tapering after an initial response (72). According to Wang and colleagues, approximately two-thirds (16/24) of patients with ICI-induced myocarditis were classified as corticosteroid-resistant type due to rebounding troponin cTnT levels during corticosteroid tapering (73). Even early- and high-dose steroids may be not sufficient in some conditions, and the mortality rate remains high in patients receiving steroids (74, 75). Conditions including continuing or aggravation of heart failure, decreased left ventricular ejection fraction, ventricular arrhythmia or complete atrioventricular block, a lack of improvement in troponin levels or the occurrence of other severe irAEs indicate additional immunosuppressive therapy (76). Therefore, effective therapeutic regimens after steroid failure have been explored. As a drug with a long history and multiple indications approved in autoimmune diseases, infliximab has been used to treat ICI-induced myocarditis.

Since the New England Journal of Medicine first published the use of infliximab for fulminant myocarditis after immune checkpoint blockade in 2016, results from 18 patients using TNF-α antibodies for ICI-induced myocarditis have been reported (**Tables 1** and **2**) (71, 77–86). The average age of these patients was 68 years; 9 of them were male. The tumor types included melanoma, urothelial carcinoma, ovarian adenocarcinoma, renal cell carcinoma, and pulmonary adenocarcinoma. Ten received PD-1 single-agent treatment, and 8 of them received PD-1 and CTLA-4 combination therapy. Symptom onset ranged from 1 week to 4 months after the first ICI treatment, and they all manifested as grade 4 ICI-induced myocarditis. Up to 7 patients experienced myocarditis accompanied by myositis. TNF-α antibodies were generally added within 3 days after steroid failure, and the dose of 5 mg/kg was mainly adopted.

**Table 1:** Articles using anti-TNF-therapy for ICI-induced myocarditis.

Authors	Year	Patient	Age	Gender	Tumour	ICIs	Onset	Grade	Other Organs
Johnson DB et al. (71)	2016	1	63	M	Melanoma	Nivolumab+Ipilimumab	15 days	4	Myositis
Frigeri M et al. (77)	2018	2	76	F	Pulmonary adenocarcinoma	Nivolumab	7 cycles	4	N/A
Agrawal N et al. (78)	2019	3	67	M	Melanoma	Nivolumab	3 cycles	4	Optic neuritis
Saibil SD et al. (79)	2019	4	67	M	Melanoma	Nivolumab+Ipilimumab	13 days	4	Rhabdomyositis
Gallegos C et al. (80)	2019	5	47	F	Melanoma	Nivolumab+Ipilimumab	3 months	4	N/A
Shah M et al. (81)	2019	6	73	M	Urothelial carcinoma	Nivolumab+Ipilimumab	N/A	4	Myositis
Padegimas A et al. (82)	2019	7	53	F	Ovarian adenocarcinoma	Pembrolizumab	4 days	4	Neurological
		8	62	F	Renal cell carcinoma	Nivolumab	5 weeks	4	N/A
Giancaterino S et al. (83)	2020	9	88	M	Melanoma	Nivolumab	22 days	4	N/A
Zhang RS et al. (84)	2021	10	62	2M, 2F	2 melanoma,1 renal cell carcinoma,1 ovarian adenocarcinoma	3 Nivolumab,1 Pembrolizumab	N/A	4	N/A
		11					N/A	4	N/A
		12					N/A	4	N/A
		13					N/A	4	N/A
Lipe DN et al. (85)	2021	14	70	M	Urothelial carcinoma	Pembrolizumab	25 days	4	Myositis
		15	81	F	Renal cell carcinoma	Nivolumab+ipilimumab	92 days	4	Myositis
		16	66	F	Renal cell carcinoma	Nivolumab+ipilimumab	132 days	4	Myositis
		17	74	F	Melanoma	Nivolumab+ipilimumab	30 days	4	Myositis
Kadokawa Y et al. (86)	2021	18	66	M	Kidney cancer	Nivolumab+ipilimumab	34 days	4	DIC,skin

F, female; M, male. N/A, not applicable.

**Table 2:** Treatment strategies and outcomes in infliximab treated ICI-induced myocarditis patients.

Patient	Treatment strategy	Dosage	Time interval	Follow-up	irAE improvement	CV mortality	All-cause mortality
1	IV methylprednisolone 1 mg/kg for 4 days and infliximab	5mg/kg	0 day	In-hospital	N	Y	Y
2	Methylprednisolone 5mg/kg/d, plasmapheresis, IVIG 1g/kg D4, infliximab D6, D27, D39	5mg/kg	2 days	5+ months	Y	N	N
3	IV methylprednisolone 1g for 3 days, prednisone 80 mg twice daily for 5 days, 2 infliximab infusions after recurrence	N/A	N/A	4+ months	Y	N	N
4	Methylprednisone 200mg on D1, then 1000mg daily for 3 days, infliximab and IVIG D4 after progression	5mg/kg	3 days	D18	N	Y	Y
5	Methylprednisolone 500 mg iv twice daily 5 days, infliximab 2 infusions	10mg/kg	0 day	1 week	N	Y	Y
6	IV methylprednisolone 1 mg/kg twice daily with mild response, then infliximab followed by 12 rounds of plasmapheresis, and subsequent IVIG	N/A	A few days	19 months	Y	N	Y
7	50 mg prednisone for 1 month then progressed, 1g methylprednisolone for 3 days but recurred upon tapering, then infliximab	5mg/kg	3 days	9+ months	Y	N	N
8	IV methylprednisolone 1mg/kg worsened, then 2g methylprednisolone for 3 days and 1 dose of infliximab	5mg/kg	N/A	2 months	Y	N	Y
9	Prednisone 40mg D1-4, methylprednisolone 125mg D5-6, 1g D7, then infliximab D9	5mg/kg	9 days	15 days	N	Y	Y
10	Pulse dose steroids, then infliximab	5mg/kg	3 days	1 year	Y	N	N
11	Pulse dose steroids, then infliximab	5mg/kg	3 days	3 months	N/A	N	Y
12	Pulse dose steroids, then infliximab	5mg/kg	1 year	1 year	Y	N	N
13	Pulse dose steroids, then infliximab	5mg/kg	3 days	3 months	N/A	N	Y
14	High-dose glucocorticoids (1-2 mg/kg) then infliximab	N/A	N/A	5 days	N/A	N/A	Y
15	High-dose glucocorticoids (1-2 mg/kg) then infliximab	N/A	N/A	In-hospital	N/A	N	N
16	High-dose glucocorticoids (1-2 mg/kg) then infliximab	N/A	N/A	In-hospital	N/A	N	N
17	High-dose glucocorticoids (1-2 mg/kg) then infliximab	N/A	N/A	26 days	N/A	N/A	Y
18	IV prednisolone: D34-36 80mg; Methylprednisolone: D37-39 1000mg; D40-44 80mg; Infliximab: D40/D54 425mg	5mg/kg	6 days	1 month	Y	N	N

IV, intravenous; IVIG, intravenous immune globulin; N/A, not applicable.

During the average follow-up of 123.2 days, 10 patients died, and 4 of them died of cardiovascular causes. Three of the 4 patients underwent autopsy, and pathological findings revealed T cells and macrophage infiltration and accumulation in the myocardium tissue. Among the 12 patients for whom the irAE status was reported after treatment, 8 of them experienced an improvement in ICI-induced myocarditis (66.7%). In all patients with available echocardiographic data, the EF was significantly lower than 50%, and these patients should be classified as having moderate-to-severe heart failure, but anti-TNF-therapy was still adopted. In a previous study by Cautela and colleagues, the authors raised a concern that infliximab was associated with a greater risk of cardiovascular death (50%) than other intensified immunosuppressive therapies (19%) when first-line steroid treatment failed (76). However, the present reported cardiovascular mortality rate of 22.2% (4/18) was not higher than the rate of 19% in a larger population. Therefore, the existing evidence is not sufficient to conclude that TNF-α inhibitors would accelerate or worsen heart failure in ICI-induced myocarditis. In the Dutch Melanoma Treatment Registry, the survival advantage to irAE was abrogated when anti-TNF is administered for steroid-refractory toxicity, mainly in the context of colitis (median overall survival 17 months in anti-TNF ± steroids versus 27 months in steroids only). Second-line immunosuppression other than anti-TNF was also used, but the median overall survival was not reported and compared (87). Therefore, it is hard to say whether the shortened survival is due to over-inhibition of immune activation against tumor growth, or negative effects solely caused by anti-TNF therapy. Furthermore, only steroids is not enough in severe life-threatening ICI-induced myocarditis.

Comparison between the patients who finally survived and died from ICI-induced myocarditis progression or other causes was shown in **Table 3**. Although the ejection fraction was within the range of heart failure with mid-range ejection fraction (HFmrEF) in all three group, the troponin level was significantly higher in patients who died (1,539 ng/L in survival group, 14,202 ng/L in all-cause mortality group, 21,461 ng/L in cardiovascular mortality group), indicating more severe cardiac injury in these patients. The onset time of symptoms after initiation of ICI was shorter in patients who died than in patients who survived (33 days versus 79 days). In different studies, the median time to symptom onset after ICI usage ranges from 16 to 65 days (88). The early onset of myocarditis might be related to the more severely damaged myocardium. Concurrent myocarditis and myositis is commonly observed after ICI treatment. In a study of 60 patients with ICI-induced myocarditis, up to 46.7% (28/60) had myositis, and 21.7% (13/60) had myasthenia gravis (MG) concurrently (76). Additionally, among patients with MG, the most common neuromuscular irAE, 16.2% and 8.8% of patients had accompanying myositis and myocarditis, respectively. Furthermore, the presence of all 3 toxicities was associated with a significantly higher risk of death (62.5%) than myocarditis alone (33.3%) (89). Similarly, 71.4% (5/7) of 7 patients with concurrent ICI-induced myositis and myocarditis who were treated with TNF-α inhibitors died, a value that is higher than 45.5% (5/10) of patients with myocarditis alone. Previous research identified combination therapy of anti-PD-1 with anti-CTLA-4 as a risk factor for ICI-induced myocarditis (66). In the TNF-α-treated patients, 75% who died of cardiovascular events received combination therapy compared with 50% of patients who survived. But the present sample size is too small to perform a convincing statistical analysis.

**Table 3:** Comparison between survivors and non-survivors.

	Survival (n=8, 44.4%)	All-cause mortality (n=10, 55.6%)	Cardiovascular mortality (n=4, 22.2%)
Average overall survival, days	216.7	89.1	11.8
Age, years	66.6	66.8	66.3
Male, %	33.3	62.5	75
CVD risk factors, n	2	2	1
Troponin, ng/L	1,539	14,202	21,461
Ejection fraction, %	40	44	46.5
Steroid pulse therapy, %	62.5	50	50
VT, n	2	1	0
Complete AV block, n	2	4	2
Cardiogenic shock, n	1	4	1
PD-1+CTLA-4 combination, %	50	62.5	75
Onset time, days	78.7	32.9	35
Myositis, n	2	5	2
Other organs involvement, n	3	0	0
Time before anti-TNF, days	3.5	4.5	3
High dose infliximab, n	0	1	1

AV, atrioventricular; CTLA-4, cytotoxic T-lymphocyte-associated protein-4; CVD, cardiovascuar disease; PD-1, programmed death-1; TNF, tumor necrosis factor.

In addition to second-line treatment in patients in whom steroid failed, anti-TNF-therapy was also proposed to be used earlier in first-line treatment or even for the prevention of ICI-induced cardiotoxicity. Michel and colleagues documented that TNF-α blockade may prevent the detrimental effect of anti-PD-1 therapy while preserving the anticancer efficacy in tumor-bearing mice (90). In 2021, Bermas and colleagues proposed a new perspective in the journal Circulation that a new and more aggressive treatment paradigm for ICI-induced myocarditis should be developed based on past experience in treating RA (70). In patients with RA, the use of more aggressive DMARDs early in the disease course, instead of starting with nonsteroidal anti-inflammatory drugs, substantially improved RA outcomes (91). In patients with grade 4 myocarditis, the authors recommended initiating therapy with pulse steroids and plasmapheresis and adding biological agents upfront as first-line treatment (70). Two patients initiated infliximab upfront, with no gap between pulse steroids and TNF-α therapy, without waiting for the efficacy of steroids. However, they both died shortly after treatment (71, 80). A 63-year-old man had profound ST-segment depression, intraventricular conduction delay, myocarditis and myositis 15 days after nivolumab treatment. Although intravenous methylprednisolone at 1g for 4 days, and infliximab at 5 mg/kg were given in a timely and simultaneous manner, the patient developed a complete heart block and died of cardiac arrest in the hospital (71). The other 47-year-old woman developed supraventricular tachycardia 3 months after nivolumab treatment, but it was ignored. One month later, she developed severe myocarditis and heart failure and was treated with 1 g of intravenous methylprednisolone for 5 days, together with 10 mg/kg infliximab for 2 days. However, her clinical course was complicated by cardiogenic shock, and she finally palliated and died in the hospital (80). Considering the high mortality rate of ICI-induced myocarditis, the treatment paradigm should undoubtedly be improved, but further studies are needed to determine whether the above modification is appropriate.

In summary, although infliximab does not increase cardiovascular risk in RA patients, the current evidence is not strong enough to support neither the conclusion that infliximab is also safe in steroid-resistant and steroid-refractory ICI-induced myocarditis, nor it will accelerate or worsen heart failure. Further studies regarding TNF-α inhibitors are necessary. If the concern about its cardiovascular risk can not be solved, other newly developed immunosupressive biologic agents should be investigated more in the future.

### 2.6 Biologic Agents of Other Targets in ICI-Induced Myocarditis

#### CTLA-4 Agonists

CTLA-4 and CD28 are homologous receptors expressed by T cells, sharing CD80/CD86, a pair of ligands expressed on the surface of antigen presenting cells (APCs). However, they mediate opposing functions in T cell activation. CD28 mediates T cell co-stimulation, while CTLA-4 serves to inhibit T cell responses (92). Abatacept, a CTLA-4 immunoglobulin fusion protein, binds to CD80/CD86 on APCs and leads to T cell anergy (93). Its close relationship with the mechanisms of immunotherapy makes it likely to reverse pathways activated by ICI, and become a promising candidate for steroid-refractory ICI-induced myocarditis.

In mouse models of ICI-induced myocarditis, the intervention of abatacept led to a reduction in cardiac immune activation and an increase in survival (94). Cases reporting the administration of abatacept in ICI-induced myocarditis have been published in recent years. It was first used in a 66-year-old woman with metastatic lung cancer, who had concurrent myositis and myocarditis after ICI treatment, and failed to respond to highdose methylprednisolone and plasmapheresis. After the administration of abatacept 500mg every 2 weeks, for a total of 5 doses, her troponin T level rapidly decreased from 6,000 ng/L and symptoms of myocarditis and myositis progressively decreased (95). Another 57-year-old renal cell carcinoma male also had concurrent myositis and myocarditis after PD-1 and CTLA-4 treatment. Despite high-dose glucocorticoids, the ICI-induced myositis and myocarditis worsened. Abatacept and mycophenolate mofetil resulted in normalized troponin I, decreased ventricular tachycardia, and alleviated symptoms (96). Additionally, a 25-year-old thymoma patient had cardiogenic shock after pembrolizumab treatment. Methylprednisolone 1g/d and addition of mycophenolate-mofetil had limited efficacy. Initiation of abatacept and ruxolitinib reversed the disease course and the patient fully recovered clinically, with ejection fraction restoring to 60% (97).

Therefore, use of abatacept in ICI-induced myocarditis is worth expecting, especially considering its efficacy in concurrent myositis and myocarditis. However, the risk of tumor relapse should also be noted. Two patients experienced tumor relapse 3 months and 4 months after abatacept treatment, respectively (96, 97). The duration of follow-up was only 1 month in another patient, the possibility of tumor relapse could not be excluded (94). Like other immunosuppressant, further research is needed to assess the optimal drug mix, dosage and duration of abatacept, in order to mitigate the potential lethality associated with irAEs while preserving antitumor beneficial effects. CD86 receptor occupancy saturation might be a reference for the timing and dosage of abatacept (97). Two prospective clinical trials have been underway to assess the safety and efficacy of abatacept in ICI-induced myocarditis (NCT05335928 and NCT05195645).

#### 2.6.2 IL-6 Receptor Inhibitors

IL-6 is recognized as another proinflammatory cytokine that is produced in the pathogenesis of various inflammatory diseases. Formation of the IL-6/IL-6R/gp130 hexamer leads to the initiation of Janus kinase (JAK)/signal transducers and activators of transcription (STAT) signaling, a pathway involved in many crucial biological processes, including cell proliferation, differentiation, apoptosis, and immune regulation (98, 99). IL-6-mediated STAT3 activation in the tumor microenvironment and cancer cells is associated with tumor cell proliferation, angiogenesis and metastasis (100, 101). In addition to the JAK/STAT pathway, IL-6 can induce the differentiation of pathogenic T_H_17 from naive T cells, and inhibit the Foxp3^+^ regulatory T cells (102). Tocilizumab is a humanized monoclonal antibody with a high affinity for human IL-6 (103). In 2017, tocilizumab was approved by the FDA to treat cytokine release syndrome (CRS) after chimeric antigen receptor T-cell (CAR-T) immunotherapy, another immune disorder occurring after T-cell activation (104, 105). Additionally, its efficacy against irAEs has been preliminarily documented. The first case was a 74-year-old woman who suffered from sustained polymorphic ventricular tachycardia, elevated N-terminal pro-B type natriuretic peptide (NT-proBNP) and cardiac troponin I (cTnI) levels, and an impaired ejection fraction (EF) of 40%, despite treated with steroid pulse therapy and intravenous immunoglobulin therapy. Tocilizumab was administered at a dose of 8 mg/kg; 4 weeks later, her cTnI level decreased to normal, ventricular rhythm transferred to sinus rhythm, and EF recovered to 60% (106). The other 57-year-old male patient experienced complete atrioventricular block although steroid pulse therapy was administered. After the tocilizumab injection, his symptoms, inflammatory biomarkers, and cardiac biomarkers significantly improved (107). For irAEs with other organ involvement, tocilizumab also produced satisfying results. According to a systemic review, 91 patients had used tocilizumab for various irAEs as of 2021. Among these patients, 76 had irAE outcomes, and up to 87% (66/76) of them experienced irAE improvements (108). Considering its excellent performance and safety profile, tocilizumab might be a potential treatment option for ICI-induced myocarditis with more evidence available.

#### 2.6.3 CD52 Antibody

CD52 is expressed on peripheral mature immune cells, mainly on T and B lymphocytes, while it is expressed at lower levels on innate immune cells. Its antibody alemtuzumab leads to antibody-dependent cell-mediated cytolysis, complement-mediated destruction, and apoptosis of these cells (109). Alemtuzumab, a CD52 antibody, is recommended in current guidelines for the treatment of ICI-induced myocarditis. However, so far treatment with alemtuzumab has only been reported in one case. In a 71-year-old woman, alemtuzumab achieved rapid resolution of ICI-induced cardiac immune toxic effects after she failed to respond to pulse methylprednisolone, mycophenolate mofetil, plasmapheresis, and rituximab therapies (110). Meanwhile, the risk of infection, malignancies and autoimmune disorders associated with alemtuzumab-induced immune reconstitution should not be ignored (111, 112).

#### 2.6.4 JAK Inhibitors

JAK inhibitors on the market are mainly small molecules rather than biologics (113). JAKs are cytoplasmic tyrosine kinases that phosphorylate tyrosine residues either on themselves or on adjacent molecules such as STAT. The JAK family has 4 members (JAK1, JAK2, JAK3, and TYK2), and the STAT family consists of 7 members. The JAK/STAT pathway mediates the effects of a broad range of molecules, including ILs, IFNs, colony-stimulating factors, growth factors and hormones (114, 115). Therefore, JAK blockade will lead to strong inhibition of the immune system, and the efficacy of JAK inhibitors is widely recognized. Studies of their effects on ICI-induced myocarditis have been performed. Tofacitinib was used in 11 patients with corticosteroid-resistant ICI-associated myocarditis patients: 7 recovered, 2 died of cardiac symptom progression, and 2 died of infection (73). Another 2 patients with ICI-induced myocarditis also recovered and were discharged after tofacitinib treatment (116).

However, if the drug is not selective for certain JAK members, safety issues might be present. For instance, in the postmarketing ORALSurveillance trial conducted by Pfizer, the JAK1/3 inhibitor tofacitinib was associated with a higher incidence of cardiovascular events and malignancies than TNF-α inhibitors (NCT02092467). Cardiovascular risk seems to be the Achilles’ heel of JAK inhibitors, and it has received great attention from regulatory agencies. Meanwhile, JAK loss-of-function mutations were found responsible for primary and acquired resistance to anti-PD-1 therapy in patients with solid tumor. IFNγ binds to the interferon gamma receptor (IFNGR1/IFNGR2) and activates downstream signaling *via* JAK1/2, finally resulting in an upregulation of MHC-I molecules and PD-L1 to the cancer cell outer membrane. JAK mutations, causing a loss-of-function phenotype, can lead to the absence of PD-L1 expression and subsequent inefficacy of anti-PD-1/PD-L1 therapy (117, 118). Therefore, JAK inhibitors should be used with caution in ICI-induced myocarditis. In the future, the roles of different JAK family members in ICI-induced myocarditis must be further clarified, and inhibitors with better selectivity are warranted. JAK inhibitors should be manipulated better to suppress its pro-tumorigenic behavior and enhance its anti-tumorigenic aspects, such as inhibiting tumor cell survival, proliferation and invasion (119).

#### 2.6.5 Other Untargeted Immunosuppressive Agents

Other untargeted immunosuppressive agents, such as antithymocyte globulin (ATG), intravenous immunoglobulin (IVIG), and mycophenolate are also recommended by current guidelines for ICI-induced myocarditis, but are not the main focus of this review. They are empirical strategies logically recommended by the experts, since the histological lesions observed in ICI-induced myocarditis are similar to those observed during acute cardiac transplant cell rejection. Several case reports with favorable outcomes support the use of them in ICI-induced myocarditis (96, 120, 121). In a pooled analysis of intensified immunosuppressive therapies for ICI-induced myocarditis, neither ATG nor IVIG showed advantage in overall survival (OR 5.4 and 1.5 for ATG and IVIG, respectively). The all-cause mortality rate and cardiovascular mortality rate were 16.6% (1/6) and 0% (0/6) for mycophenolate (76). But the number of cases were still too limited to reach a convincing conclusion. More studies of them are needed, especially in combination with steroids in first-line treatment and with other biologic agents in second-line steroid-refractory patients.

## 3 Ongoing Clinical Trials

Researchers have not clearly determined which biologic antagonist would be optimized for ICI-related myocarditis, especially in steroid-resistant or steroid-refractory patients. According to the United States National Institutes of Health registry ClinicalTrials.gov, several trials are underway and will be informative (**Table 4**). Recently in April 2022, a randomized, double-blind, and placebo-controlled study was registered to evaluate the efficacy and safety of abatacept. The trial is a phase 3 study with a sample size of 390 patients. It is estimated to be completed in 5 years (NCT05335928). Similarly, another phase 2 trial initiated in March 2022 aims to assess different doses of abatacept in ICI-induced myocarditis (NCT05195645). A trial initiated in 2016 aimed to compare the efficacy of the TNF-α inhibitor infliximab plus prednisone with methylprednisolone plus prednisone in individuals with ICI-induced diarrhoea; however, it was withdrawn in 2018 due to insufficient enrolment (NCT02763761). The IL-6 inhibitor tocilizumab is being investigated as a treatment for steroid-dependent irAEs to identify the percentage of participants who discontinue steroid treatment (NCT04375228). In another arm of this trial, the CD20 antibody rituximab was used. In addition to T cell activation, ICIs activate B cells *via* regulatory T cells (122). Rituximab has shown certain efficacy in B cell-dependent irAEs such as MG and Sjogren syndrome (123), and this trial may provide more insights into its efficacy against other irAEs. Another trial of the JAK inhibitor tofacitinib in patients with immune-related colitis who failed to respond to corticosteroids is ongoing and aims to investigate the clinical remission of diarrhoea (NCT04768504). CD24Fc treatment attenuates inflammation associated with viral infections, autoimmunity, and graft-versus-host diseases (124). A comparison of the efficacy of CD24Fc with a placebo in treating irAEs is also being investigated (NCT04552704).

**Table 4:** Ongoing registered clinical trials.

Identifier	Inclusion	Arm	Sample size	Primary outcome	Status	Start date	Completion date
NCT02763761	Immune Related Diarrhea	Arm A: Infliximab + Prednisone	N/A	Proportion of responders to less than or equal to grade 1	Withdrawn	16-Aug	17-Mar
		Arm B: Methylprednisolone + Prednisone					
NCT04552704	Immune related adverse events	Arm A: CD24Fc	78	Incidence of new adverse event; recovery rate; time to recovery from grade 2 or 3	Active, not recruiting	20-Oct	22-Feb
		Arm B: Placebo					
NCT04375228	Steroid-Dependent immune related adverse events	Arm A: Rituximab	30	Percentage of paticipants to discontinue steroid treatment	Not yet recruiting	21-Jun	24-Feb
		Arm B: Tocilizumab					
NCT04768504	Refractory immune-related Colitis	Arm A: Tofacitinib	10	Clinical Remission of Diarrhea	Recruiting	21-Nov	23-Sep
NCT05195645	Severe or corticosteroid-resistant ICI-myocarditis	Arms A-C: 10mg/kg, 20mg/kg and 25mg/kg abatacept	21	Proportion of CD86 receptor occupancy saturation ≥ 80%	Not yet recruiting	22-Mar	24-Sep
NCT05335928	ICI-induced myocarditis	Arm A: Abatacept plus	390	Major adverse cardiac events	Not yet recruiting	22-May	27-Apr
		Arm B: Placebo					

N/A, not applicable; ICI, immune checkpoint inhibitor.

## 4 Summary

Although the risk of aggravating heart failure is a potential safety concern, infliximab has been used in patients with ICI-induced myocarditis with more reported cases than other biologic agents (**Table 1**). It helps more patients with grade 4 cardiac irAEs to achieve an improvement and survive (**Table 2**). The association between TNF-α inhibitors and progressive heart failure does not seem to be relevant in patients with ICI-induced myocarditis. But the current evidence is not strong enough to draw a solid conclusion, further studies are warranted. More severe myocardium injury after ICI treatment might be a predictor related to a poor prognosis, despite the use of anti-TNF-therapy (**Table 3**). If the cardiovascular risk of TNF-α inhibitors in ICI-induced myocarditis can not be eliminated, other biologic agents, such as abatacept, tocilizumab and alemtuzumab are promising, while data for their effects on ICI-induced myocarditis are still limited. Clinical trials including CTLA-4 agonists, anti-IL-6, anti-CD24, anti-CD20 therapies and JAK inhibitors are expected to provide more insights into whether better interventions exist other than the TNF-α inhibitors currently recommended in the guidelines.

## Author Contributions

XL collected materials and wrote the paper. WW, YL, and WC provided the idea and reviewed the manuscript. LF, YL, and WC helped with the final revision of the paper. All authors contributed to the article and approved the submitted version.

## Funding

This work was supported by the Beijing Natural Science Foundation [grant number 7192156], the Capital’s Funds for Health Improvement and Research, CFH [grant number 2020-2-40110], and the CAMS Innovation Fund for Medical Sciences [grant number CIFMS,2020-I2M-C&T-B-006] to WC; and the National Natural Science Foundation of China [grant number 82000470] to YL.

## Conflict of Interest

The authors declare that the research was conducted in the absence of any commercial or financial relationships that could be construed as a potential conflict of interest.

## Publisher’s Note

All claims expressed in this article are solely those of the authors and do not necessarily represent those of their affiliated organizations, or those of the publisher, the editors and the reviewers. Any product that may be evaluated in this article, or claim that may be made by its manufacturer, is not guaranteed or endorsed by the publisher.

## References

[1] Galluzzi L Humeau J Buqué A Zitvogel L Kroemer G. Immunostimulation With Chemotherapy in the Era of Immune Checkpoint Inhibitors. Nat Rev Clin Oncol (2020) 17(12):725–41. doi: 10.1038/s41571-020-0413-z 32760014

[2] Maruhashi T Sugiura D Okazaki IM Okazaki T. LAG-3: From Molecular Functions to Clinical Applications. J Immunother Cancer (2020) 8(2):e001014. doi: 10.1136/jitc-2020-001014 32929051 PMC7488795

[3] Harjunpää H Guillerey C. TIGIT as an Emerging Immune Checkpoint. Clin Exp Immunol (2020) 200(2):108–19. doi: 10.1111/cei.13407 PMC7160651 31828774

[4] Tian T Li Z. Targeting Tim-3 in Cancer With Resistance to PD-1/PD-L1 Blockade. Front Oncol (2021) 11:731175. doi: 10.3389/fonc.2021.731175 34631560 PMC8492972

[5] Ning Z Liu K Xiong H. Roles of BTLA in Immunity and Immune Disorders. Front Immunol (2021) 12:654960. doi: 10.3389/fimmu.2021.654960 33859648 PMC8043046

[6] Zhang W Huang Q Xiao W Zhao Y Pi J Xu H. Advances in Anti-Tumor Treatments Targeting the CD47/Sirpα Axis. Front Immunol (2020) 11:18. doi: 10.3389/fimmu.2020.00018 32082311 PMC7003246

[7] Postow MA Sidlow R Hellmann MD. Immune-Related Adverse Events Associated With Immune Checkpoint Blockade. N Engl J Med (2018) 378(2):158–68. doi: 10.1056/NEJMra1703481 29320654

[8] Frayberg M Yung A Zubiri L Zlotoff DA Reynolds KL. What the Cardiologist Needs to Know About Cancer Immunotherapies and Complications. Curr Treat Options Oncol (2021) 22(6):53. doi: 10.1007/s11864-021-00844-1 34037918

[9] Mahmood SS Fradley MG Cohen JV Nohria A Reynolds KL Heinzerling LM. Myocarditis in Patients Treated With Immune Checkpoint Inhibitors. J Am Coll Cardiol (2018) 71(16):1755–64. doi: 10.1016/j.jacc.2018.02.037 PMC6196725 29567210

[10] Salem JE Manouchehri A Moey M Lebrun-Vignes B Bastarache L Pariente A. Cardiovascular Toxicities Associated With Immune Checkpoint Inhibitors: An Observational, Retrospective, Pharmacovigilance Study. Lancet Oncol (2018) 19(12):1579–89. doi: 10.1016/S1470-2045(18)30608-9 PMC6287923 30442497

[11] Schneider BJ Naidoo J Santomasso BD Lacchetti C Adkins S Anadkat M. Management of Immune-Related Adverse Events in Patients Treated With Immune Checkpoint Inhibitor Therapy: ASCO Guideline Update. J Clin Oncol (2021) 39(36):4073–126. doi: 10.1200/JCO.21.01440 34724392

[12] Haanen JBAG Carbonnel F Robert C Kerr KM Peters S Larkin J. Management of Toxicities From Immunotherapy: ESMO Clinical Practice Guidelines for Diagnosis, Treatment and Follow-Up. Ann Oncol (2017) 28(suppl_4):iv119–42. doi: 10.1093/annonc/mdx225 28881921

[13] Brahmer JR Abu-Sbeih H Ascierto PA Brufsky J Cappelli LC Cortazar FB. Society for Immunotherapy of Cancer (SITC) Clinical Practice Guideline on Immune Checkpoint Inhibitor-Related Adverse Events. J Immunother Cancer (2021) 9(6):e002435. doi: 10.1136/jitc-2021-002435 34172516 PMC8237720

[14] National Comprehensive Cancer Network. NCCN Guidelines Management of Immunotherapy-Related Toxicities (2022). Available at: https://www.nccn.org/professionals/physician_gls/pdf/immunotherapy (Accessed May 28, 2022).

[15] Jang DI Lee AH Shin HY Song HR Park JH Kang TB. The Role of Tumor Necrosis Factor Alpha (TNF-α) in Autoimmune Disease and Current TNF-α Inhibitors in Therapeutics. Int J Mol Sci (2021) 22(5):2719. doi: 10.3390/ijms22052719 33800290 PMC7962638

[16] Miossec P Kolls JK. Targeting IL-17 and TH17 Cells in Chronic Inflammation. Nat Rev Drug Discov (2012) 11(10):763–76. doi: 10.1038/nrd3794 23023676

[17] Hunter CA Jones SA. IL-6 as a Keystone Cytokine in Health and Disease. Nat Immunol (2015) 16(5):448–57. doi: 10.1038/ni1117-1271a 25898198

[18] Harb H Chatila TA. Mechanisms of Dupilumab. Clin Exp Allergy (2020) 50(1):5–14. doi: 10.1111/cea.13491 31505066 PMC6930967

[19] Aggarwal BB Gupta SC Kim JH. Historical Perspectives on Tumor Necrosis Factor and its Superfamily: 25 Years Later, a Golden Journey. Blood (2012) 119(3):651–65. doi: 10.1182/blood-2011-04-325225 PMC3265196 22053109

[20] Moss ML Jin SL Milla ME Bickett DM Burkhart W Carter HL. Cloning of a Disintegrin Metalloproteinase That Processes Precursor Tumor-Necrosis Factor-Alpha. Nature (1997) 385(6618):733–6. doi: 10.1038/385733a0 9034191

[21] Palladino MA Bahjat FR Theodorakis EA Moldawer LL. Anti-TNF-Alpha Therapies: The Next Generation. Nat Rev Drug Discov (2003) 2(9):736–46. doi: 10.1038/nrd1175 12951580

[22] Wajant H Pfizenmaier K Scheurich P. Tumor Necrosis Factor Signaling. Cell Death Differ (2003) 10(1):45–65. doi: 10.1038/sj.cdd.4401189 12655295

[23] Rolski F Błyszczuk P. Complexity of TNF-α Signaling in Heart Disease. J Clin Med (2020) 9(10):3267. doi: 10.3390/jcm9103267 PMC7601316 33053859

[24] Hsu H Shu HB Pan MG Goeddel DV. TRADD-TRAF2 and TRADD-FADD Interactions Define Two Distinct TNF Receptor 1 Signal Transduction Pathways. Cell (1996) 84(2):299–308. doi: 10.1016/s0092-8674(00)80984-8 8565075

[25] Yang S Wang J Brand DD Zheng SG. Role of TNF-TNF Receptor 2 Signal in Regulatory T Cells and Its Therapeutic Implications. Front Immunol (2018) 9:784. doi: 10.3389/fimmu.2018.00784 29725328 PMC5916970

[26] Borghi A Verstrepen L Beyaert R. TRAF2 Multitasking in TNF Receptor-Induced Signaling to NF-κb, MAP Kinases and Cell Death. Biochem Pharmacol (2016) 116:1–10. doi: 10.1016/j.bcp.2016.03.009 26993379

[27] Guo X Yin H Li L Chen Y Li J Doan J. Cardioprotective Role of Tumor Necrosis Factor Receptor-Associated Factor 2 by Suppressing Apoptosis and Necroptosis. Circulation (2017) 136(8):729–42. doi: 10.1161/CIRCULATIONAHA.116.026240 PMC5568494 28572508

[28] Rossol M Meusch U Pierer M Kaltenhäuser S Häntzschel H Hauschildt S. Interaction Between Transmembrane TNF and TNFR1/2 Mediates the Activation of Monocytes by Contact With T Cells. J Immunol (2007) 179(6):4239–48. doi: 10.4049/jimmunol.179.6.4239 17785864

[29] Ardestani S Deskins DL Young PP. Membrane TNF-Alpha-Activated Programmed Necrosis Is Mediated by Ceramide-Induced Reactive Oxygen Species. J Mol Signal (2013) 8(1):12. doi: 10.1186/1750-2187-8-12 24180579 PMC3895838

[30] Carswell EA Old LJ Kassel RL Green S Fiore N Williamson B. An Endotoxin-Induced Serum Factor That Causes Necrosis of Tumors. Proc Natl Acad Sci U S A (1975) 72(9):3666–70. doi: 10.1073/pnas.72.9.3666 PMC433057 1103152

[31] Pennica D Nedwin GE Hayflick JS Seeburg PH Derynck R Palladino MA. Human Tumor Necrosis Factor: Precursor Structure, Expression and Homology to Lymphotoxin. Nature (1984) 312(5996):724–9. doi: 10.1038/312724a0 6392892

[32] Brouckaert PG Leroux-Roels GG Guisez Y Tavernier J Fiers W. *In Vivo* Anti-Tumor Activity of Recombinant Human and Murine TNF, Alone and in Combination With Murine IFN-Gamma, on a Syngeneic Murine Melanoma. Int J Cancer (1986) 38(5):763–9. doi: 10.1002/ijc.2910380521 3095251

[33] Kettelhut IC Fiers W Goldberg AL. The Toxic Effects of Tumor Necrosis Factor *In Vivo* and Their Prevention by Cyclooxygenase Inhibitors. Proc Natl Acad Sci USA (1987) 84(12):4273–7. doi: 10.1073/pnas.84.12.4273 PMC305067 3108890

[34] Lienard D Ewalenko P Delmotte JJ Renard N Lejeune FJ. High-Dose Recombinant Tumor Necrosis Factor Alpha in Combination With Interferon Gamma and Melphalan in Isolation Perfusion of the Limbs for Melanoma and Sarcoma. J Clin Oncol (1992) 10(1):52–60. doi: 10.1200/JCO.1992.10.1.52 1727926

[35] de Wilt JH Manusama ER van Tiel ST van Ijken MG ten Hagen TL Eggermont AM. Prerequisites for Effective Isolated Limb Perfusion Using Tumor Necrosis Factor Alpha and Melphalan in Rats. Br J Cancer (1999) 80(1-2):161–6. doi: 10.1038/sj.bjc.6690335 PMC2362986 10389992

[36] Balkwill F. Tumor Necrosis Factor and Cancer. Nat Rev Cancer (2009) 9(5):361–71. doi: 10.1038/nrc2628 19343034

[37] Seynhaeve AL Hoving S Schipper D Vermeulen CE Ga dW-A ST vT. Tumor Necrosis Factor Alpha Mediates Homogeneous Distribution of Liposomes in Murine Melanoma That Contributes to a Better Tumor Response. Cancer Res (2007) 67(19):9455–62. doi: 10.1158/0008-5472.CAN-07-1599 17909055

[38] Moore RJ Owens DM Stamp G Arnott C Burke F East N. Mice Deficient in Tumor Necrosis Factor-Alpha Are Resistant to Skin Carcinogenesis. Nat Med (1999) 5(7):828–31. doi: 10.1038/10552 10395330

[39] Pikarsky E Porat RM Stein I Abramovitch R Amit S Kasem S. NF-kappaB Functions as a Tumor Promoter in Inflammation-Associated Cancer. Nature (2004) 431(7007):461–6. doi: 10.1038/nature02924 15329734

[40] Komori J Marusawa H Machimoto T Endo Y Kinoshita K Kou T. Activation-Induced Cytidine Deaminase Links Bile Duct Inflammation to Human Cholangiocarcinoma. Hepatology (2008) 47(3):888–96. doi: 10.1002/hep.22125 18306229

[41] Hagemann T Wilson J Kulbe H Li NF Leinster DA Charles K. Macrophages Induce Invasiveness of Epithelial Cancer Cells *via* NF-Kappa B and JNK. J Immunol (2005) 175(2):1197–205. doi: 10.4049/jimmunol 16002723

[42] Hagemann T Wilson J Burke F Kulbe H Li NF Plüddemann A. Ovarian Cancer Cells Polarize Macrophages Toward a Tumor-Associated Phenotype. J Immunol (2006) 176(8):5023–32. doi: 10.4049/jimmunol.176.8.5023 16585599

[43] Li B Vincent A Cates J Brantley-Sieders DM Polk DB Young PP. Low Levels of Tumor Necrosis Factor Alpha Increase Tumor Growth by Inducing an Endothelial Phenotype of Monocytes Recruited to the Tumor Site. Cancer Res (2009) 69(1):338–48. doi: 10.1158/0008-5472.CAN-08-1565 PMC2651676 19118019

[44] Wallach D. Preparations of Lymphotoxin Induce Resistance to Their Own Cytotoxic Effect. J Immunol (1984) 132(5):2464–9. 6609199

[45] Madhusudan S Foster M Muthuramalingam SR Braybrooke JP Wilner S Kaur K. A Phase II Study of Etanercept (Enbrel), a Tumor Necrosis Factor Alpha Inhibitor in Patients With Metastatic Breast Cancer. Clin Cancer Res (2004) 10(19):6528–34. doi: 10.1158/1078-0432.CCR-04-0730 15475440

[46] Madhusudan S Muthuramalingam SR Braybrooke JP Wilner S Kaur K Han C. Study of Etanercept, a Tumor Necrosis Factor-Alpha Inhibitor, in Recurrent Ovarian Cancer. J Clin Oncol (2005) 23(25):5950–9. doi: 10.1200/JCO.2005.04.127 16135466

[47] Harrison ML Obermueller E Maisey NR Hoare S Edmonds K Li NF. Tumor Necrosis Factor Alpha as a New Target for Renal Cell Carcinoma: Two Sequential Phase II Trials of Infliximab at Standard and High Dose. J Clin Oncol (2007) 25(29):4542–9. doi: 10.1200/JCO.2007.11.2136 17925549

[48] Brown ER Charles KA Hoare SA Rye RL Jodrell DI Aird RE. A Clinical Study Assessing the Tolerability and Biological Effects of Infliximab, a TNF-Alpha Inhibitor, in Patients With Advanced Cancer. Ann Oncol (2008) 19(7):1340–6. doi: 10.1093/annonc/mdn054 18325912

[49] Darnell EP Mooradian MJ Baruch EN Yilmaz M Reynolds KL. Immune-Related Adverse Events (irAEs): Diagnosis, Management, and Clinical Pearls. Curr Oncol Rep (2020) 22(4):39. doi: 10.1007/s11912-020-0897-9 32200442

[50] Melsheimer R Geldhof A Apaolaza I Schaible T. Remicade® (Infliximab): 20 Years of Contributions to Science and Medicine. Biologics (2019) 13:139–78. doi: 10.2147/BTT.S207246 PMC6679695 31440029

[51] Levine B Kalman J Mayer L Fillit HM Packer M. Elevated Circulating Levels of Tumor Necrosis Factor in Severe Chronic Heart Failure. N Engl J Med (1990) 323(4):236–41. doi: 10.1056/NEJM199007263230405 2195340

[52] Meldrum DR. Tumor Necrosis Factor in the Heart. Am J Physiol (1998) 274(3):R577–95. doi: 10.1152/ajpregu.1998.274.3.R577 9530222

[53] Deswal A Bozkurt B Seta Y Parilti-Eiswirth S Hayes FA Blosch C. Safety and Efficacy of a Soluble P75 Tumor Necrosis Factor Receptor (Enbrel, Etanercept) in Patients With Advanced Heart Failure. Circulation (1999) 99(25):3224–6. doi: 10.1161/01.cir.99.25.3224 10385494

[54] Mann DL McMurray JJ Packer M Swedberg K Borer JS Colucci WS. Targeted Anticytokine Therapy in Patients With Chronic Heart Failure: Results of the Randomized Etanercept Worldwide Evaluation (RENEWAL). Circulation (2004) 109(13):1594–602. doi: 10.1161/01.CIR.0000124490.27666.B2 15023878

[55] Chung ES Packer M Lo KH Fasanmade AA Willerson JT Anti-TNF Therapy Against Congestive Heart Failure Investigators. Randomized, Double-Blind, Placebo-Controlled, Pilot Trial of Infliximab, a Chimeric Monoclonal Antibody to Tumor Necrosis Factor-Alpha, in Patients With Moderate-to-Severe Heart Failure: Results of the Anti-TNF Therapy Against Congestive Heart Failure (ATTACH) Trial. Circulation (2003) 107(25):3133–40. doi: 10.1161/01.CIR.0000077913.60364.D2 12796126

[56] Asgeri M Pourafkari L Kundra A Javadzadegan H Negargar S Nader ND. Dual Effects of Tumor Necrosis Factor Alpha on Myocardial Injury Following Prolonged Hypoperfusion of the Heart. Immunol Invest (2015) 44(1):23–35. doi: 10.3109/08820139.2014.921689 24949667

[57] Aderka D Engelmann H Maor Y Brakebusch C Wallach D. Stabilization of the Bioactivity of Tumor Necrosis Factor by Its Soluble Receptors. J Exp Med (1992) 175(2):323–9. doi: 10.1084/jem.175.2.323 PMC2119112 1310100

[58] Scallon BJ Moore MA Trinh H Knight DM Ghrayeb J. Chimeric Anti-TNF-Alpha Monoclonal Antibody Ca2 Binds Recombinant Transmembrane TNF-Alpha and Activates Immune Effector Functions. Cytokine (1995) 7(3):251–9. doi: 10.1006/cyto.1995.0029 7640345

[59] Choy E Ganeshalingam K Semb AG Szekanecz Z Nurmohamed M. Cardiovascular Risk in Rheumatoid Arthritis: Recent Advances in the Understanding of the Pivotal Role of Inflammation, Risk Predictors and the Impact of Treatment. Rheumatol (Oxford) (2014) 53(12):2143–54. doi: 10.1093/rheumatology/keu224 PMC4241890 24907149

[60] Wolfe F Michaud K. Heart Failure in Rheumatoid Arthritis: Rates, Predictors, and the Effect of Anti-Tumor Necrosis Factor Therapy. Am J Med (2004) 116(5):305–11. doi: 10.1016/j.amjmed.2003.09.039 14984815

[61] Listing J Strangfeld A Kekow J Schneider M Kapelle A Wassenberg S. Does Tumor Necrosis Factor Alpha Inhibition Promote or Prevent Heart Failure in Patients With Rheumatoid Arthritis? Arthritis Rheum (2008) 58(3):667–77. doi: 10.1002/art.23281 18311816

[62] Jacobsson LT Turesson C Gülfe A Kapetanovic MC Petersson IF Saxne T. Treatment With Tumor Necrosis Factor Blockers Is Associated With a Lower Incidence of First Cardiovascular Events in Patients With Rheumatoid Arthritis. J Rheumatol (2005) 32(7):1213–8. 15996054

[63] Dixon WG Watson KD Lunt M Hyrich KL British Society for Rheumatology Biologics Register Control Centre Consortium Silman AJ. Reduction in the Incidence of Myocardial Infarction in Patients With Rheumatoid Arthritis Who Respond to Anti-Tumor Necrosis Factor Alpha Therapy: Results From the British Society for Rheumatology Biologics Register. Arthritis Rheum (2007) 56(9):2905–12. doi: 10.1002/art.22809 PMC2435427 17763428

[64] Liao KP. Cardiovascular Disease in Patients With Rheumatoid Arthritis. Trends Cardiovasc Med (2017) 27(2):136–40. doi: 10.1016/j.tcm.2016.07.006 PMC5253086 27612551

[65] Khunger A Battel L Wadhawan A More A Kapoor A Agrawal N. New Insights Into Mechanisms of Immune Checkpoint Inhibitor-Induced Cardiovascular Toxicity. Curr Oncol Rep (2020) 22(7):65. doi: 10.1007/s11912-020-00925-8 32514647

[66] Moslehi J Lichtman AH Sharpe AH Galluzzi L Kitsis RN. Immune Checkpoint Inhibitor-Associated Myocarditis: Manifestations and Mechanisms. J Clin Invest (2021) 131(5):e145186. doi: 10.1172/JCI145186 PMC7919710 33645548

[67] Cohen A Barlesi F Ederhy S Thuny F. Clinical Features, Management, and Outcomes of Immune Checkpoint Inhibitor-Related Cardiotoxicity. Circulation (2017) 136(21):2085–7. doi: 10.1161/CIRCULATIONAHA 29158217

[68] Nishimura H Nose M Hiai H Minato N Honjo T. Development of Lupus-Like Autoimmune Diseases by Disruption of the PD-1 Gene Encoding an ITIM Motif-Carrying Immunoreceptor. Immunity (1999) 11(2):141–51. doi: 10.1016/s1074-7613(00)80089-8 10485649

[69] Nishimura H Okazaki T Tanaka Y Nakatani K Hara M Matsumori A. Autoimmune Dilated Cardiomyopathy in PD-1 Receptor-Deficient Mice. Science (2001) 291(5502):319–22. doi: 10.1126/science.291.5502.319 11209085

[70] Bermas BL Zaha VG. Mending Broken Hearts: A New Treatment Paradigm for Immune Checkpoint Inhibitor-Induced Myocarditis. Circulation (2021) 143(8):767–9. doi: 10.1161/CIRCULATIONAHA 33617309

[71] Johnson DB Balko JM Compton ML Chalkias S Gorham J Xu Y. Fulminant Myocarditis With Combination Immune Checkpoint Blockade. N Engl J Med (2016) 375(18):1749–55. doi: 10.1056/NEJMoa1609214 PMC5247797 27806233

[72] Beattie J Rizvi H Fuentes P Luo J Schoenfeld A Lin IH. Success and Failure of Additional Immune Modulators in Steroid-Refractory/Resistant Pneumonitis Related to Immune Checkpoint Blockade. J Immunother Cancer (2021) 9(2):e001884. doi: 10.1136/jitc-2020-001884 33568350 PMC7878154

[73] Wang C Lin J Wang Y Hsi DH Chen J Liu T. Case Series of Steroid-Resistant Immune Checkpoint Inhibitor Associated Myocarditis: A Comparative Analysis of Corticosteroid and Tofacitinib Treatment. Front Pharmacol (2021) 12:770631. doi: 10.3389/fphar.2021.770631 34938185 PMC8685452

[74] Lehmann LH Cautela J Palaskas N Baik AH Meijers WC Allenbach Y. Clinical Strategy for the Diagnosis and Treatment of Immune Checkpoint Inhibitor–Associated Myocarditis: A Narrative Review. JAMA Cardiol (2021) 6(11):1329–37. doi: 10.1001/jamacardio.2021.2241 34232253

[75] Zhang L Zlotoff DA Awadalla M Mahmood SS Nohria A Hassan MZO. Major Adverse Cardiovascular Events and the Timing and Dose of Corticosteroids in Immune Checkpoint Inhibitor-Associated Myocarditis. Circulation (2020) 141(24):2031–4. doi: 10.1161/CIRCULATIONAHA PMC7301778 32539614

[76] Cautela J Zeriouh S Gaubert M Bonello L Laine M Peyrol M. Intensified Immunosuppressive Therapy in Patients With Immune Checkpoint Inhibitor-Induced Myocarditis. J Immunother Cancer (2020) 8(2):e001887. doi: 10.1136/jitc-2020-001887 33298621 PMC7725077

[77] Frigeri M Meyer P Banfi C Giraud R Hachulla AL Spoerl D. Immune Checkpoint Inhibitor-Associated Myocarditis: A New Challenge for Cardiologists. Can J Cardiol (2018) 34(1):92.e1–3. doi: 10.1016/j.cjca.2017.09.025 29275889

[78] Agrawal N Khunger A Vachhani P Colvin TA Hattoum A Spangenthal E. Cardiac Toxicity Associated With Immune Checkpoint Inhibitors: Case Series and Review of the Literature. Case Rep Oncol (2019) 12(1):260–76. doi: 10.1159/000498985 PMC6465686 31011325

[79] Saibil SD Bonilla L Majeed H Sotov V Hogg D Chappell MA. Fatal Myocarditis and Rhabdomyositis in a Patient With Stage IV Melanoma Treated With Combined Ipilimumab and Nivolumab. Curr Oncol (2019) 26(3):e418–21. doi: 10.3747/co.26.4381 PMC6588051 31285688

[80] Gallegos C Rottmann D Nguyen VQ Baldassarre LA. Myocarditis With Checkpoint Inhibitor Immunotherapy: Case Report of Late Gadolinium Enhancement on Cardiac Magnetic Resonance With Pathology Correlate. Eur Heart J Case Rep (2019) 3(1):yty149. doi: 10.1093/ehjcr/yty149 31020225 PMC6439394

[81] Shah M Tayar JH Abdel-Wahab N Suarez-Almazor ME. Myositis as an Adverse Event of Immune Checkpoint Blockade for Cancer Therapy. Semin Arthritis Rheum (2019) 48(4):736–40. doi: 10.1016/j.semarthrit.2018.05.006 29909921

[82] Padegimas A Agarwal P Fleitman J Carver J Rao S Mather P. Case Series of Ventricular Tachycardia and Myocarditis From Programmed Cell-Death Protein-1 Inhibitor Treated With Infliximab. JACC Clin Electrophysiol (2019) 5(8):989–92. doi: 10.1016/j.jacep.2019.05.001 31439303

[83] Giancaterino S Abushamat F Duran J Lupercio F DeMaria A Hsu JC. Complete Heart Block and Subsequent Sudden Cardiac Death From Immune Checkpoint Inhibitor-Associated Myocarditis. HeartRhythm Case Rep (2020) 6(10):761–4. doi: 10.1016/j.hrcr.2020.07.015 PMC7573344 33101950

[84] Zhang RS Padegimas A Murphy KM Evans PT Peters CJ Domenico CM. Treatment of Corticosteroid Refractory Immune Checkpoint Inhibitor Myocarditis With Infliximab: A Case Series. Cardiooncology (2021) 7(1):13. doi: 10.1186/s40959-021-00095-x 33785062 PMC8008661

[85] Lipe DN Galvis-Carvajal E Rajha E Wechsler AH Gaeta S. Immune Checkpoint Inhibitor-Associated Myasthenia Gravis, Myositis, and Myocarditis Overlap Syndrome. Am J Emerg Med (2021) 46:51–5. doi: 10.1016/j.ajem.2021.03.005 33721590

[86] Kadokawa Y Takagi M Yoshida T Tatsumi A Fujita K Inoue T. Efficacy and Safety of Infliximab for Steroid-Resistant Immune-Related Adverse Events: A Retrospective Study. Mol Clin Oncol (2021) 14(4):65. doi: 10.3892/mco.2021.2227 33680456 PMC7890436

[87] Verheijden RJ May AM Blank CU Aarts MJB van den Berkmortel FWPJ van den Eertwegh AJM. Association of Anti-TNF With Decreased Survival in Steroid Refractory Ipilimumab and Anti-PD1-Treated Patients in the Dutch Melanoma Treatment Registry. Clin Cancer Res (2020) 26(9):2268–74. doi: 10.1158/1078-0432.CCR-19-3322 31988197

[88] Matzen E Bartels LE Løgstrup B Horskær S Stilling C Donskov F. Immune Checkpoint Inhibitor-Induced Myocarditis in Cancer Patients: A Case Report and Review of Reported Cases. Cardiooncology (2021) 7(1):27. doi: 10.1186/s40959-021-00114-x 34365980 PMC8351114

[89] Johnson DB Manouchehri A Haugh AM Quach HT Balko JM Lebrun-Vignes B. Neurologic Toxicity Associated With Immune Checkpoint Inhibitors: A Pharmacovigilance Study. J Immunother Cancer (2019) 7(1):134. doi: 10.1186/s40425-019-0617-x 31118078 PMC6530194

[90] Michel L Helfrich I Hendgen-Cotta UB Mincu RI Korste S Mrotzek SM. Targeting Early Stages of Cardiotoxicity From Anti-PD1 Immune Checkpoint Inhibitor Therapy. Eur Heart J (2022) 43(4):316–29. doi: 10.1093/eurheartj/ehab430 34389849

[91] van Vollenhoven RF. Treatment of Rheumatoid Arthritis: State of the Art 2009. Nat Rev Rheumatol (2009) 5(10):531–41. doi: 10.1038/nrrheum.2009.182 19798027

[92] Rowshanravan B Halliday N Sansom DM. CTLA-4: A Moving Target in Immunotherapy. Blood (2018) 131(1):58–67. doi: 10.1182/blood-2017-06-741033 29118008 PMC6317697

[93] Ingelfinger JR Schwartz RS. Immunosuppression–the Promise of Specificity. N Engl J Med (2005) 353(8):836–9. doi: 10.1056/NEJMe058166 16120865

[94] Wei SC Meijers WC Axelrod ML Anang NAS Screever EM Wescott EC. A Genetic Mouse Model Recapitulates Immune Checkpoint Inhibitor-Associated Myocarditis and Supports a Mechanism-Based Therapeutic Intervention. Cancer Discov (2021) 11(3):614–25. doi: 10.1158/2159-8290.CD-20-0856 PMC8041233 33257470

[95] Salem JE Allenbach Y Vozy A Brechot N Johnson DB Moslehi JJ. Abatacept for Severe Immune Checkpoint Inhibitor-Associated Myocarditis. N Engl J Med (2019) 380(24):2377–9. doi: 10.1056/NEJMc1901677 31189043

[96] Jespersen MS Fanø S Stenør C Møller AK. A Case Report of Immune Checkpoint Inhibitor-Related Steroid-Refractory Myocarditis and Myasthenia Gravis-Like Myositis Treated With Abatacept and Mycophenolate Mofetil. Eur Heart J Case Rep (2021) 5(11):ytab342. doi: 10.1093/ehjcr/ytab342 34870082 PMC8637790

[97] Nguyen LS Bretagne M Arrondeau J Zahr N Ederhy S Abbar B. Reversal of Immune-Checkpoint Inhibitor Fulminant Myocarditis Using Personalized-Dose-Adjusted Abatacept and Ruxolitinib: Proof of Concept. J Immunother Cancer (2022) 10(4):e004699. doi: 10.1136/jitc-2022-004699 35383117 PMC8984056

[98] Garbers C Aparicio-Siegmund S Rose-John S. The IL-6/Gp130/STAT3 Signaling Axis: Recent Advances Towards Specific Inhibition. CurrOpin Immunol (2015) 34:75–82. doi: 10.1016/j.coi.2015.02.008 25749511

[99] Hong DS Angelo LS Kurzrock R. Interleukin-6 and Its Receptor in Cancer: Implications for Translational Therapeutics. Cancer (2007) 110(9):1911–28. doi: 10.1002/cncr.22999 17849470

[100] Kitamura H Ohno Y Toyoshima Y Ohtake J Homma S Kawamura H. Interleukin-6/STAT3 Signaling as a Promising Target to Improve the Efficacy of Cancer Immunotherapy. Cancer Sci (2017) 108(10):1947–52. doi: 10.1111/cas.13332 PMC5623748 28749573

[101] Bharti R Dey G Mandal M. Cancer Development, Chemoresistance, Epithelial to Mesenchymal Transition and Stem Cells: A Snapshot of IL-6 Mediated Involvement. Cancer Lett (2016) 375(1):51–61. doi: 10.1016/j.canlet.2016.02.048 26945971

[102] Bettelli E Carrier Y Gao W Korn T Strom TB Oukka M. Reciprocal Developmental Pathways for the Generation of Pathogenic Effector TH17 and Regulatory T Cells. Nature (2006) 441(7090):235–8. doi: 10.1038/nature04753 16648838

[103] Sebba A. Tocilizumab: The First Interleukin-6-Receptor Inhibitor. Am J Health Syst Pharm (2008) 65(15):1413–8. doi: 10.2146/ajhp070449 18653811

[104] Lee DW Gardner R Porter DL Louis CU Ahmed N Jensen M. Current Concepts in the Diagnosis and Management of Cytokine Release Syndrome. Blood (2014) 124(2):188–95. doi: 10.1182/blood-2014-05-552729 PMC4093680 24876563

[105] Le RQ Li L Yuan W Shord SS Nie L Habtemariam BA. FDA Approval Summary: Tocilizumab for Treatment of Chimeric Antigen Receptor T Cell-Induced Severe or Life-Threatening Cytokine Release Syndrome. Oncologist (2018) 23(8):943–7. doi: 10.1634/theoncologist.2018-0028 PMC6156173 29622697

[106] Wang H Tian R Gao P Wang Q Zhang L. Tocilizumab for Fulminant Programmed Death 1 Inhibitor-Associated Myocarditis. J Thorac Oncol (2020) 15(3):e31–2. doi: 10.1016/j.jtho.2019.09.080 32093854

[107] Doms J Prior JO Peters S Obeid M. Tocilizumab for Refractory Severe Immune Checkpoint Inhibitor-Associated Myocarditis. Ann Oncol (2020) 31(9):1273–5. doi: 10.1016/j.annonc.2020.05.005 PMC7229714 32425357

[108] Campochiaro C Farina N Tomelleri A Ferrara R Lazzari C De Luca G. Tocilizumab for the Treatment of Immune-Related Adverse Events: A Systematic Literature Review and a Multicentre Case Series. Eur J Intern Med (2021) 93:87–94. doi: 10.1016/j.ejim.2021.07.016 34391591

[109] Syed YY. Alemtuzumab: A Review in Relapsing Remitting Multiple Sclerosis. Drugs (2021) 81(1):157–68. doi: 10.1007/s40265-020-01437-2 33367970

[110] Esfahani K Buhlaiga N Thébault P Lapointe R Johnson NA Miller WH Jr. Alemtuzumab for Immune-Related Myocarditis Due to PD-1 Therapy. N Engl J Med (2019) 380(24):2375–6. doi: 10.1056/NEJMc1903064 31189042

[111] Ruiz-Camps I Aguilar-Company J. Risk of Infection Associated With Targeted Therapies for Solid Organ and Hematological Malignancies. Ther Adv Infect Dis (2021) 8:2049936121989548. doi: 10.1177/2049936121989548 33680453 PMC7897815

[112] Vakrakou AG Tzanetakos D Evangelopoulos ME Fragoulis GE Kazakou P Lekka E. IgG4-Related Autoimmune Manifestations in Alemtuzumab-Treated Multiple Sclerosis Patients. J Neuroimmunol (2021) 361:577759. doi: 10.1016/j.jneuroim.2021.577759 34742035

[113] Favoino E Prete M Catacchio G Ruscitti P Navarini L Giacomelli R. Working and Safety Profiles of JAK/STAT Signaling Inhibitors. Are These Small Mol Also Smart? Autoimmun Rev (2021) 20(3):102750. doi: 10.1016/j.autrev.2021.102750 33482338

[114] Fragoulis GE McInnes IB Siebert S. New Players in the Field of Immune-Mediated Diseases, Beyond Rheumatoid Arthritis. Rheumatol (Oxford) (2019) 58(Suppl 1):i43–54. doi: 10.1093/rheumatology/key276 PMC6390879 30806709

[115] Villarino AV Kanno Y O'Shea JJ. Mechanisms and Consequences of Jak-STAT Signaling in the Immune System. Nat Immunol (2017) 18(4):374–84. doi: 10.1038/ni.3691 PMC11565648 28323260

[116] Liu Y Jiang L. Tofacitinib for Treatment in Immune-Mediated Myocarditis: The First Reported Cases. J Oncol Pharm Pract (2020) 11:1078155220947141. doi: 10.1177/1078155220947141 32781887

[117] Marabelle A Aspeslagh S Postel-Vinay S Soria JC. JAK Mutations as Escape Mechanisms to Anti-PD-1 Therapy. Cancer Discov (2017) 7(2):128–30. doi: 10.1158/2159-8290.CD-16-1439 28167612

[118] Shin DS Zaretsky JM Escuin-Ordinas H Garcia-Diaz A Hu-Lieskovan S Kalbasi A. Primary Resistance to PD-1 Blockade Mediated by JAK1/2 Mutations. Cancer Discov (2017) 7(2):188–201. doi: 10.1158/2159-8290.CD-16-1223 27903500 PMC5296316

[119] Johnson DE O'Keefe RA Grandis JR. Targeting the IL-6/JAK/STAT3 Signalling Axis in Cancer. Nat Rev Clin Oncol (2018) 15(4):234–48. doi: 10.1038/nrclinonc.2018.8 PMC5858971 29405201

[120] Tay RY Blackley E McLean C Moore M Bergin P Gill S. Successful Use of Equine Anti-Thymocyte Globulin (ATGAM) for Fulminant Myocarditis Secondary to Nivolumab Therapy. Br J Cancer (2017) 117(7):921–4. doi: 10.1038/bjc.2017.253 PMC5625667 28797029

[121] Balanescu DV Donisan T Palaskas N Lopez-Mattei J Kim PY Buja LM. Immunomodulatory Treatment of Immune Checkpoint Inhibitor-Induced Myocarditis: Pathway Toward Precision-Based Therapy. Cardiovasc Pathol (2020) 47:107211. doi: 10.1016/j.carpath.2020.107211 32268262

[122] Hollern DP Xu N Thennavan A Glodowski C Garcia-Recio S Mott KR. B Cells and T Follicular Helper Cells Mediate Response to Checkpoint Inhibitors in High Mutation Burden Mouse Models of Breast Cancer. Cell (2019) 179(5):1191–206.e21. doi: 10.1016/j.cell.2019.10.028 31730857 PMC6911685

[123] Verma N Jaffer M Pina Y Peguero E Mokhtari S. Rituximab for Immune Checkpoint Inhibitor Myasthenia Gravis. Cureus (2021) 13(7):e16337. doi: 10.7759/cureus.16337 34395120 PMC8357079

[124] Song NJ Allen C Vilgelm AE Riesenberg BP Weller KP Reynolds K. Treatment With Soluble CD24 Attenuates COVID-19-Associated Systemic Immunopathology. J Hematol Oncol (2022) 15(1):5. doi: 10.1186/s13045-021-01222-y 35012610 PMC8744064

